# The Role of Real‐Time Engagement in Shaping Social Media Check‐In Behavior: Moderating Effects of Trust and Peer Influence

**DOI:** 10.1002/brb3.70887

**Published:** 2025-09-21

**Authors:** Xiaoshuang Lu, Kavitha Balakrishnan, Tak Jie Chan, Meng Na

**Affiliations:** ^1^ Faculty of Applied Communication Multimedia University Cyberjaya Malaysia; ^2^ Graduate School of Business Universiti Kebangsaan Malaysia Bangi Selangor Malaysia

**Keywords:** check‐in behavior, information credibility, peer influence, real‐time engagement, social media, trust

## Abstract

Social media platforms have reached a transformative stage, blending traditional digital interactions with real‐time engagement features to foster user participation and connectivity. As real‐time engagement mechanisms such as interactive communication, personalized feedback, and dynamic content recommendations become integral, they significantly shape users’ digital behaviors, including venue check‐ins. Drawing upon Trust Theory, Social Influence Theory, and the Elaboration Likelihood Model (ELM), this study investigates how real‐time engagement factors influence users’ check‐in behavior on social media, emphasizing perceived credibility as a mediating factor and exploring the moderating roles of Trust in Platforms and peer influence (PI). Using survey data collected from 500 active social media users in China, analyzed through structural equation modeling, this research identifies significant pathways by which personal trust anchors (PTAs), information density value (IDV), and peer consensus dynamics (PCDs) collectively affect check‐in decisions. The findings reveal that trust significantly facilitates the translation of engagement into immediate actions, whereas PI and perceived information authenticity enhance credibility perceptions under conditions of real‐time interaction. This research advances the literature by integrating previously isolated constructs—trust, information quality, and peer validation—to present a comprehensive understanding of real‐time engagement's role in digital participation. Practically, the study provides strategic insights for social media marketers and platform developers, emphasizing the critical balance of interactivity, trust mechanisms, and social validation to optimize user engagement within real‐time digital ecosystems.

## Graphical abstract

This graphical abstract illustrates how real‐time engagement factor supported by trust and peer influence‐ impact user check in behavior on social media through the mediating role of perceived credibility.

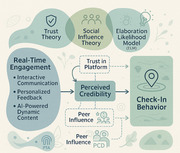

## 1 Introduction

The contemporary digital ecosystem is rapidly evolving, with real‐time engagement emerging as a defining characteristic of user interaction across social media and location‐based services (LBS). Moving beyond passive browsing, digital participation is increasingly shaped by AI‐driven personalization, instantaneous interactivity, and dynamic peer validation mechanisms. In this environment, check‐in behavior (CIB)—where users voluntarily share their real‐time locations—has become a widespread mode of engagement, providing platforms with rich behavioral data and enabling users to signal identity, status, and belonging (Anderson et al. 2025; Hui et al. 2024). The global LBS market, valued at $62 billion in 2023, is expected to double by 2028 (Fortune Business Insights 2024), and China has emerged as a global epicenter of this transformation.

Platforms, such as WeChat, Xiaohongshu (RED), Meituan, and Dianping, have deeply embedded real‐time check‐in features into everyday digital life, blending social sharing, location tagging, and consumer reviews into seamless user experiences. In particular, China's mobile‐first, app‐centric culture—fueled by urban density, super‐app ecosystems, and high digital literacy—has accelerated the normalization of location‐based participation (Zhou 2023). Yet, despite this behavioral ubiquity, empirical research has lagged in unpacking the psychological and social drivers behind Chinese users’ real‐time CIB. Prior studies have largely focused on macro‐level patterns (e.g., urban mobility, consumer loyalty) or functional motivations (Hwang et al. 2024; Xu et al. 2021) but have neglected the micro‐level interplay of trust, information credibility, and peer dynamics that shape these spontaneous disclosures—particularly in high‐stakes, high‐surveillance digital environments like China's.

This study identifies three specific gaps. First, although *trust* has been widely studied in domains such as mobile payments and electronic word‐of‐mouth (eWOM) (Ismagilova et al. 2020; Talwar et al. 2020), its role in shaping instantaneous and location‐revealing actions such as check‐ins—especially in data‐sensitive contexts like China—remains critically underexplored. Chinese users navigate a complex digital terrain where platform reliability, algorithmic transparency, and state surveillance intersect. Thus, the extent to which interpersonal trust (e.g., in influencers or content creators) and institutional trust (e.g., in platforms like RED or WeChat) motivate check‐ins is a pressing question for both theory and practice.

Second, the ambivalent role of peer influence (PI) warrants deeper scrutiny. Social endorsement cues—such as trending check‐ins, algorithmic popularity badges, and influencer‐generated heat maps—are central to Chinese digital culture. Studies report that 64% of global users act more readily when peers endorse content (Roth et al. 2021), but in China, this number may be even higher due to collectivist values and the normalization of social comparison (Ding et al. 2024). However, excessive consensus or uniformity of opinion may trigger skepticism or perceived manipulation, especially among younger Chinese users increasingly sensitive to inauthentic content (Ye and Zhao 2023). Thus, the double‐edged nature of peer validation in high‐pressure, reputation‐driven digital spaces like Xiaohongshu remains theoretically underdeveloped.

Third, information credibility remains a pivotal yet under‐theorized construct in the context of real‐time location sharing. Although credible and authentic content is known to foster trust (Muda and Hamzah 2021), Chinese users often exhibit selective skepticism toward both platform‐mediated and user‐generated content (UGC) due to prior scandals involving fake reviews, ghost followers, or algorithmic bias (Liu, He et al. 2024; Wang, Zhang et al. 2025). Furthermore, research shows that although 72% of Chinese users report trusting online reviews, only about 50%–55% act on them (Anderson 2021). This behavioral gap suggests that credibility alone is insufficient and may need to be mediated by trust or moderated by peer cues to trigger actual engagement. The Elaboration Likelihood Model (ELM) (Petty and Cacioppo 1986) offers a relevant theoretical lens to explain how users process these cues cognitively, but applications of ELM in Chinese check‐in contexts remain limited (Moradi and Zihagh 2022; Wang, Huang et al. 2025).

To address these empirical and theoretical gaps, this study integrates Trust Theory, Social Influence Theory, and the ELM into a unified framework that explains Chinese users’ CIB on digital platforms. Specifically, it examines:

- How real‐time engagement features (e.g., AI personalization, interactivity, content responsiveness) shape CIB.
- The moderating roles of Trust in Platform (TP) and PI within the cultural and technological context of Chinese digital platforms.
- The mediating role of Perceived Credibility (PC) in converting cognitive engagement into behavioral participation.

Utilizing partial least squares structural equation modeling (PLS‐SEM) on empirical data from a diverse sample of digital users in China, this study delivers context‐sensitive insights into how trust, credibility, and social cues interact to shape real‐time digital behavior. Its contributions are threefold: (a) offering a contextualized theoretical advancement by applying integrated models to Chinese digital ecosystems, (b) revealing the boundary conditions of trust and PI in high‐density information environments, and (c) providing actionable design and policy implications for platforms operating in China's hyper‐competitive, algorithmically mediated digital space.

As China continues to shape global digital trends—particularly in platform integration, influencer economies, and AI‐driven content ecosystems—understanding the psychosocial mechanisms behind behaviors like check‐ins becomes vital. This research not only bridges a critical scholarly gap but also offers culturally grounded, empirically validated strategies for optimizing engagement, credibility, and trust in real‐time digital interaction.

## 2 Literature Review

### 2.1 Real‐Time Engagement and CIB

The existing literature extensively explores real‐time engagement, trust, and social influence within digital platforms, particularly in social media and LBSs. Real‐time engagement, characterized by immediate interactions, instantaneous feedback, and dynamic user experiences, has become a significant driver of digital user participation (Khamaj and Ali 2024). Recent research underscores that interactivity, timely responses, and personalized content significantly enhance user involvement by fostering emotional engagement and perceived authenticity, which directly influence behaviors like real‐time location check‐ins (Sang et al. 2024). However, prior studies frequently examine real‐time interactions in isolation, rarely integrating them comprehensively with trust and PI to explain CIB fully.

Trust emerges consistently as a critical determinant influencing users’ willingness to share their location information in real time. Building upon Trust Theory (Mayer et al. 1995), researchers highlight that perceived security, transparency, and reliability of platforms significantly affect users’ comfort and willingness to engage (Ahmed and Aziz 2024). For example, Singhal et al. (2024) found that transparent privacy policies, data security assurances, and trustworthy verification systems substantially increase users’ trust levels, subsequently enhancing their likelihood of sharing real‐time check‐ins. Nonetheless, existing studies predominantly focus on general online trust, leaving the specific impact of trust on immediate, impulsive behaviors like real‐time check‐ins less understood.

Social Influence Theory (Kelman 1958) further explains user behavior, highlighting peer validation and social norms as influential factors. Recent studies (Aziz et al. 2025; Chang and Chen 2014; van Binh et al. 2023) confirm that peer‐driven social signals, such as friend endorsements, recommendations, and social proof, significantly increase users’ propensity to engage in location‐sharing activities. Such validation reinforces perceived authenticity and motivates conformity to group behaviors, making users more likely to participate when others within their social network have already done so. However, although PI has been extensively studied concerning general online behaviors, few have explicitly explored how peer dynamics influence real‐time decisions in check‐in contexts, particularly when integrated with trust and PC.

This study addresses these critical gaps by integrating real‐time engagement, trust dynamics, and PI into a cohesive theoretical framework specifically tailored for CIB on digital platforms. Unlike prior research that examines these dimensions separately, this study investigates their interconnected effects, emphasizing trust and PI as moderators shaping the relationship between real‐time engagement and immediate user check‐in decisions. This comprehensive approach offers novel insights, particularly within the unique context of Chinese social media users.

### 2.2 Theoretical Underpinning

This study integrates Trust Theory, Social Influence Theory, and the ELM to construct a comprehensive framework explaining CIB on social media platforms. These theoretical perspectives provide a multidimensional approach to understanding how trust, PI, and cognitive information processing shape user engagement, credibility perceptions, and real‐time participation. Unlike the previous research, which often examines these factors in isolation, this study synthesizes these perspectives to capture their dynamic interplay in digital ecosystems, where algorithmic mediation and social validation play crucial roles in shaping user behavior.

Trust Theory (Mayer et al. 1995) serves as a foundational framework, positing that trust emerges from three key dimensions: ability, benevolence, and integrity. Extensively applied in diverse digital contexts—ranging from AI decision‐making (Solberg et al. 2020) to e‐commerce trust dynamics (Tang et al. 2021)—the theory underscores trust's pivotal role in reducing uncertainty and fostering user engagement. Although prior studies have demonstrated trust's influence on behaviors such as eWOM sharing (Ismagilova et al. 2020) and mobile payment adoption (Talwar et al. 2020), its specific impact on real‐time CIBs remains underexplored. This study extends Trust Theory by introducing two critical dimensions:

1. Personal trust anchors (PTAs)—Representing familiarity, transparency, and consistency in digital interactions, PTA fosters interpersonal trust by enhancing user confidence in check‐in disclosures.
2. Institutional trust (TP)—Capturing confidence in platform governance, security, and reliability, institutional trust moderates user engagement by mitigating privacy concerns and reinforcing credibility in location‐sharing decisions.

Although Trust Theory provides insights into trust formation, Social Influence Theory (Kelman 1958) offers a compelling explanation of how peer consensus and social norms shape user engagement in real‐time digital contexts. This theory conceptualizes social influence as occurring through compliance, identification, and internalization, making it a powerful determinant of digital participation. Research on mobile learning adoption (Nie et al. 2020), online health communities (Zhou 2021), and social media interactions (MUĞAN 2020) confirms the strong impact of social validation on behavioral conformity. However, its application to peer‐driven CIBs remains incomplete, particularly in examining how social validation mechanisms—such as trending check‐ins, influencer endorsements, and algorithmic recommendations—interact with trust dynamics to amplify engagement.

This study builds on Social Influence Theory by introducing peer consensus dynamics (PCDs) as a key construct, encapsulating how perceived social validation, network conformity, and peer endorsements influence PC and CIB. Although prior research indicates that 64% of users engage more actively when content receives peer endorsements (Nie et al. 2020), excessive peer reliance may undermine credibility and perceived authenticity (Höttecke and Allchin 2020). This dual role of PI—as both an engagement driver and credibility disruptor—requires further empirical validation, particularly in socially dynamic, real‐time environments.

The ELM (Petty and Cacioppo 1986) complements these theories by explaining how users process information under varying conditions of motivation and ability. Applied in diverse contexts such as booking intentions in online travel (Leong et al. 2017), mHealth adoption (Guo et al. 2020), and eWOM credibility (Putra and Suprapti 2020), ELM identifies two key persuasion routes:

1. Central route processing—Engaged when users actively evaluate information credibility, depth, and richness, making high‐quality content (information density value—IDV) a crucial determinant of PC.
2. Peripheral route processing—Driven by heuristic cues, social validation, and trust signals, this route suggests that PTAs and PCD can trigger CIBs even in the absence of deep cognitive processing.

Despite ELM's established role in digital engagement research, its application to real‐time social media interactions remains limited. This study extends ELM by linking information credibility mechanisms to real‐time engagement behaviors, exploring how trust and social influence interact with cognitive processing strategies to determine user participation in check‐in activities.

**FIGURE 1:**
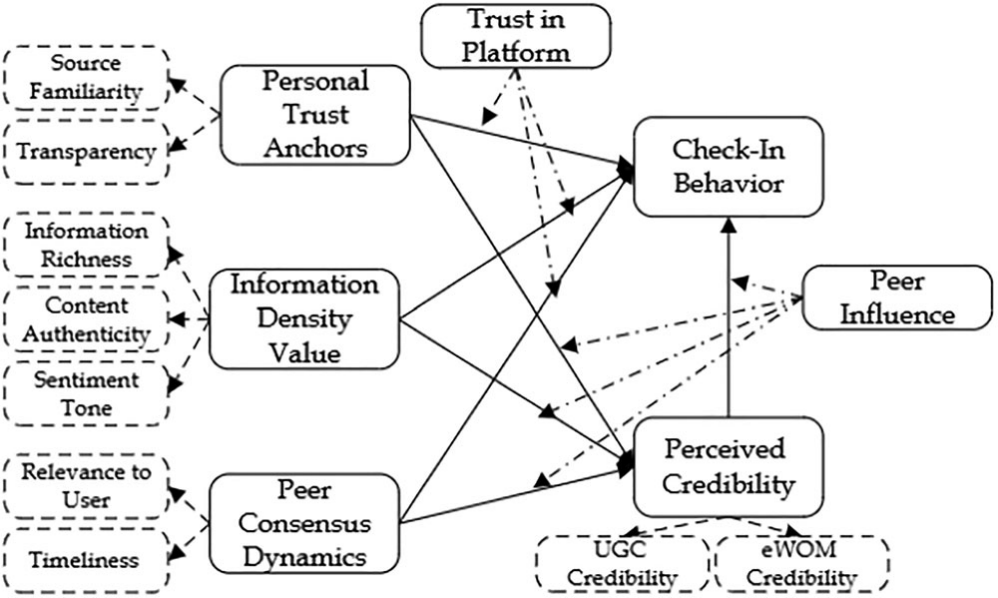
Research framework.

By integrating Trust Theory, Social Influence Theory, and ELM, this study constructs a holistic framework (Figure 1) for understanding real‐time engagement in social media CIBs. This approach not only clarifies the mechanisms underpinning trust formation, social validation, and cognitive processing but also offers empirical insights into how these factors dynamically interact to shape user decisions in evolving digital ecosystems. The findings will contribute to both theoretical advancement and practical recommendations, guiding platform designers, marketers, and policymakers in fostering trust‐based, socially engaging, and cognitively optimized digital environments that sustain user participation.

### 2.3 Hypothesis Development

#### 2.3.1 Antecedents of CIB

PTAs play a pivotal role in reducing uncertainty and encouraging engagement on social media platforms. These anchors—such as verified accounts, familiar digital identities, or influencers—leverage both cognitive trust (based on rational evaluation) and emotional trust (based on interpersonal warmth) to create a sense of security (Komiak and Benbasat 2006; Yan et al. 2024). Trust diminishes the psychological cost of action in uncertain digital environments, where users often face anxiety stemming from information asymmetry or platform unfamiliarity (Anderson et al. 2019). Signals such as trustmarks, verified reviewer profiles, or reputation badges act as heuristics that enhance platform trustworthiness and promote behaviors like check‐ins (Hughes et al. 2024). For instance, Akhtar et al. (2024) found that trust in virtual influencers significantly boosted consumer engagement, illustrating how trust anchors drive visible user behaviors.

IDV refers to the richness, depth, and relevance of UGC. High IDV enhances perceived informativeness and diagnosticity, giving users confidence in their behavioral decisions (Zhang et al. 2023; Zhu, Li et al. 2020). In uncertain contexts, dense and relevant information reduces ambiguity and risk perception, enabling users to take actions like check‐ins with greater psychological assurance (Kim et al. 2022). The inclusion of multimedia content (images, videos, emojis) and positive sentiment within reviews further amplifies the persuasive effect of the message (Batumalai 2023; Wang et al. 2023). This aligns with media richness theory, where more vivid and interactive content stimulates user responses.

PCDs refer to how the volume and alignment of peer behaviors—such as likes, comments, or past check‐ins—drive conformity through social validation. The power of normative influence becomes especially salient in digital contexts where users interpret peer consensus as a proxy for credibility and popularity (Sterman and Dogan 2015; Telzer et al. 2020). The bandwagon effect, amplified through sequential peer exposures, can significantly shape user behavior even in the absence of personal conviction (Sarkar et al. 2019). Network properties like centrality and social proximity increase the persuasive impact of influential users or communities (Hasani‐Mavriqi et al. 2018). From a reactance theory perspective, scarcity cues and the fear of missing out (FOMO) may further motivate users to engage in compensatory behaviors like check‐ins to regain a sense of control (Brock 1968; Yuen et al. 2020).

Taken together, trust cues, informational richness, and peer validation form a powerful triad influencing CIB. Therefore, the following hypothesis is proposed:

**H1**: (a) Personal trust anchors, (b) information density value, and (c) peer consensus dynamics positively influence check‐in behavior.

#### 2.3.2 Determinants of PC

PC is a critical construct in digital behavior research, especially in environments characterized by peer‐generated content and uncertain quality (Román et al. 2023). PTAs positively influence credibility evaluations by offering familiar, trustworthy, and personalized cues. As trust builds through these anchors, users are more likely to accept information as reliable and act upon it (McGloin et al. 2014). Even in instances of minor trust violations, users tend to maintain favorable credibility perceptions due to trust inertia or cognitive heuristics that protect against decision fatigue and emotional discomfort (Anderson et al. 2019).

IDV further enhances PC by presenting rich, structured, and verifiable content (Hazen et al. 2023). When users encounter detailed and authentic reviews—especially those containing contextual depth or experiential descriptions—their ability to assess message reliability improves (Liu, Lin et al. 2024). This is especially true in information‐rich decision environments like tourism, where perceived informativeness drives action (Minseong and Jihye 2019). This is consistent with expectancy theory, where users proactively seek detailed and high‐quality content to reduce anticipated negative outcomes under uncertainty (Ngunjiri 2020; Reiss 1991).

PCDs strengthen PC through collective validation. Shared agreement within a peer group signals that the information has undergone informal vetting by others, which enhances users’ cognitive confidence in the message (Koranteng et al. 2022). Influential peers or network central actors further shape perceptions by exerting social authority, which users interpret as a heuristic of credibility (Hasani‐Mavriqi et al. 2018). Familiarity with the platform and alignment with social norms contribute to how users filter, weigh, and accept information (Eg et al. 2023).

In light of these findings, the following hypothesis is developed:

**H2**: *(a) Personal trust anchors, (b) information density value, and (c) peer consensus dynamics positively influence perceived credibility*.

#### 2.3.3 Moderating Role of Platform Trust

TP functions as a contextual amplifier of user engagement by shaping how individuals interpret and act upon various trust cues in socially dense, information‐rich digital environments (Alam et al. 2025). A high degree of platform trust reduces perceived uncertainty and enhances confidence in acting on available content, thereby strengthening the behavioral impact of PTAs, IDV, and PCDs (Mao et al. 2020).

When users perceive the platform as secure and reputable, they are more likely to rely on identity cues such as verified profiles or trustmarks (Andonopoulos et al. 2023), which deepens the effect of PTA on CIB. Similarly, the presence of detailed, structured, and authentic information enhances user engagement more strongly under high platform trust, as users believe the platform maintains quality assurance mechanisms (Luo et al. 2023; Zhu, Lin, et al. 2020). Platform‐level features like consistent branding, privacy assurances, and responsive interfaces also contribute to this trust ecosystem, reducing the cognitive effort needed to evaluate credibility independently.

Importantly, platform trust can also moderate the role of peer consensus (Grüner et al. 2024). Although users may initially depend on peer validation under conditions of uncertainty, platform trust allows users to confidently act on available information without over‐relying on social cues (Otterbring and Folwarczny 2024; Pérez López et al. 2025). Drawing on trust transfer theory (Lu and Wang 2022), platform trust facilitates the transfer of interpersonal trust to system‐level trust, especially in mediated contexts where face‐to‐face cues are absent.

Thus, platform trust not only serves as a moderator of individual‐level trust cues but also reduces psychological anxiety tied to scarcity, perceived risk, or information overload—ultimately reinforcing user behaviors like check‐ins.

**H3**: *Trust in platform positively moderates the relationship between (a) personal trust anchors and check‐in behavior, (b) information density value and check‐in behavior, and (c) peer consensus dynamics and check‐in behavior*.

#### 2.3.4 Moderating Role of PI

PI significantly affects how users process digital information and evaluate the credibility of content (Ahmed and Paracha 2021), especially in uncertain or anxiety‐inducing contexts. Through mechanisms such as social norms, audience feedback, and consensus cues, PI moderates the effect of information richness (INFR) and social alignment on PC (Özdemir et al. 2023; Ozdemir et al. 2020; Reinikainen et al. 2020).

Digital cues like comments, likes, and follower counts activate bandwagon effects, signaling that a message or source has received social approval. This is especially influential in collectivist cultures or during periods of threat and scarcity, where individuals turn to group validation to guide decision‐making and alleviate emotional discomfort (Telzer et al. 2020; Wang et al. 2023). In these contexts, PI amplifies the credibility derived from rich information (e.g., visuals, in‐depth reviews) and consensus indicators (e.g., “most helpful” tags, viral sharing) (Sterman and Dogan 2015; Yu et al. 2021).

Moreover, Social Identity Theory and Social Validation Theory suggest that individuals rely more heavily on peer cues when faced with complex information or ambiguous environments. PI can thus heighten or dampen credibility assessments depending on the social context and alignment of group opinions (Argyris et al. 2021; Koranteng et al. 2022).

Taken together, PI serves as a critical moderator that strengthens the impact of both information quality and social alignment on users’ PC.

**H4**: *Peer influence positively moderates the relationship between (a) information density value and perceived credibility and (b) peer consensus dynamics and perceived credibility*.

#### 2.3.5 PC as a Mediator

PC acts as a critical psychological mechanism that translates trust‐related signals—such as trusted sources, detailed content, and peer agreement—into concrete user behaviors like check‐ins (Naab et al. 2020). When users perceive content as credible, they experience reduced uncertainty and anxiety, which facilitates behavioral responses in digital contexts (Lep et al. 2020).

PTAs, including verified reviewers or influencers, enhance credibility by reducing psychological distance and reinforcing source trustworthiness, even in the presence of minor violations (Campagna et al. 2022). IDV—rich in multimedia elements, depth, and structure—improves the perceived informativeness and reliability of content, making users feel more confident in their decision‐making (Wang et al. 2023; Zhu, Lin, et al. 2020). This supports expectancy theory, in which individuals actively seek high‐quality content to mitigate the anticipated negative consequences of uncertainty (Reiss 1991; Zhu, Li et al. 2020).

Simultaneously, PCDs offer social proof that bolsters credibility. The volume, alignment, and identity of engaged peers shape how users evaluate content, especially under conditions of scarcity or perceived risk (Jiménez‐Barreto et al. 2020). High‐status individuals and tight‐knit networks amplify this effect, reinforcing the perception that the content can be trusted and acted upon.

Given its central role in reducing uncertainty and transforming trust cues into behavioral responses, PC is posited to mediate the effects of PTA, IDV, and PCD on user engagement.

**H5**: *Perceived credibility positively mediates the relationship between (a) personal trust anchors, (b) information density value, and (c) peer consensus dynamics and check‐in behavior*.

## 3 Methodology

### 3.1 Research Design

This study employs a quantitative approach to investigate the relationships among PTAs, IDV, PCDs, TP, PI, PC, and CIB. A cross‐sectional design was adopted, and data were collected through quota sampling in China and analyzed using SmartPLS 4.0, a widely utilized tool for SEM in behavioral research (Hair et al. 2022). Similar cross‐sectional approaches have been successfully employed in studies exploring trust and user engagement on social media platforms (Mao et al. 2020).

### 3.2 Sampling

This study employed a quota sampling technique, a form of non‐probability sampling designed to ensure proportional representation across key demographic variables (Alam, Haq, et al. 2025). Quotas were established on the basis of three demographic parameters—geographic region, gender, and age group—as defined by the 2021 Chinese National Census (National Bureau of Statistics of China 2019). This approach aimed to capture the diversity of China's active social media user base, thereby enhancing the external validity of the findings.

Participants were recruited via algorithm‐assisted delivery of online questionnaires (Couper 2017; Hou et al. 2020) through three major social platforms—WeChat, Weibo, and Douyin—using a third‐party survey distribution service (e.g., Wenjuanxing). This system enabled real‐time monitoring of response rates and demographic profiles, dynamically adjusting the delivery frequency to underrepresented strata in order to fulfill the predefined quotas. The algorithm was calibrated to track participant attributes in real time and redirect outreach as needed, in accordance with best practices in digital quota sampling (Abramova et al. 2017).

Inclusion criteria required that participants (a) be Chinese nationals aged 18 or above, (b) be active users of social media, and (c) have engaged in location‐based CIB within the past 30 days. To capture behavioral variability and minimize response bias, the sample included both regular and occasional check‐in users.

A total of 500 valid responses were collected over a 4‐week period. Data privacy and anonymity were maintained in compliance with the Personal Information Protection Law (PIPL) of China. Quality control measures—including duplicate response detection and time‐based validity checks—were implemented to ensure the integrity of the dataset.

### 3.3 Measurement Items

To ensure conceptual clarity and measurement validity, this study employed reflective constructs grounded in well‐established theoretical frameworks and peer‐reviewed scales. All measurement items were adapted from validated sources and contextualized for the Chinese digital environment through rigorous translation and back‐translation procedures (Brislin 1970). Each item was rated using a 5‐point Likert scale ranging from 1 (strongly disagree) to 5 (strongly agree). The full list of measurement items and their sources is provided in Appendix A. PTA was conceptualized as a higher order construct comprising source familiarity and transparency. Source familiarity was adapted from Komiak and Benbasat (2006), whereas transparency and consistency items were based on Mao et al. (2020) and Mcknight et al. (2011), respectively. IDV was measured through three sub‐dimensions: INFR (Zhang et al. 2023), content authenticity (CA) (Zhu et al. 2020), and sentiment tone (Wang et al. 2023). PCDs reflected the user's perception of content relevance and timeliness, operationalized using scales adapted from Sarkar et al. (2019) and Hasani‐Mavriqi et al. (2018). PC was operationalized as a second‐order construct comprising UGC Credibility and eWOM Credibility, based on Zhu, Li et al. (2020) and Wang et al. (2023), respectively. TP assessed user perceptions of security, reliability, and procedural transparency, following the measurement framework proposed by Mao et al. (2020). PI focused on normative and informational influences from the user's social circle, using measurement items adapted from Reinikainen et al. (2020). Finally, CIB, the dependent variable, measured the extent and intention of users’ location‐sharing activities on social media. The items were adapted from Luarn et al. (2015), reflecting behavioral frequency and platform‐based influence. All constructs and indicators were psychometrically validated in the measurement model analysis (see Section 4.1), with detailed item wording, coding, and citations provided in Appendix A.

## 4 Results

The demographic distribution (refer to Table 1) of respondents in this study reflects a balanced and representative sample, adhering to quota sampling criteria based on geographic location, gender, age, and other relevant variables. The majority of respondents were from East China (38.0%), followed by Central China (26.0%), West China (23.0%), and Northeast China (13.0%), mirroring the population density and digital adoption trends across the regions. Gender representation was nearly equal, with males accounting for 51.0% and females for 49.0%, ensuring balanced insights into behavioral dynamics. The age distribution reveals a predominance of younger and middle‐aged respondents, with 34.0% in the 25–34 age group and 30.0% in the 35–44 range. This is consistent with the demographic profile of active social media users in China, where digital engagement peaks among these cohorts. Educational attainment is skewed toward higher education, with 52.0% holding undergraduate degrees and 22.0% having completed postgraduate studies, indicative of the educationally progressive nature of urban social media users. Occupational diversity is notable, with white‐collar workers (46.0%) forming the largest group, followed by students (24.0%), reflecting the digitally active segments of the population. Monthly income levels further support this profile, with 40.0% earning between RMB 5001 and 10,000, capturing the middle‐income segment, a key demographic for understanding digital engagement patterns. Platform usage is dominated by WeChat (62.0%), with Weibo (22.0%) and Douyin (16.0%) playing secondary roles, emphasizing the centrality of WeChat as the primary digital platform in China. Behavioral segmentation highlights that 58.0% are frequent check‐in users, with occasional users constituting 32.0%, ensuring the inclusion of varied engagement levels. Smartphone usage (87.0%) underscores the mobile‐first nature of digital interactions in China, with minimal reliance on tablets (8.0%) and desktops/laptops (5.0%). The marital status of respondents reveals a predominance of married individuals (52.0%), with single respondents at 44.0%, offering a nuanced understanding of social influences on digital behavior.

**TABLE 1:** Demographics and quota of the respondents.

Demographic variables	Categories	Frequency (*n*)	Percentage
Geographic location	East China	190	38.0
	Central China	130	26.0
	West China	115	23.0
	Northeast China	65	13.0
Gender	Male	255	51.0
	Female	245	49.0
Age group	18–24 years	100	20.0
	25–34 years	170	34.0
	35–44 years	150	30.0
	45+ years	80	16.0
Educational level	High school	130	26.0
	Undergraduate	260	52.0
	Postgraduate	110	22.0
Occupation	Student	120	24.0
	White‐collar worker	230	46.0
	Self‐employed	70	14.0
	Others	80	16.0
Monthly income (RMB)	Below 5000	160	32.0
	5001–10,000	200	40.0
	Above 10,000	140	28.0
Platform usage	WeChat	310	62.0
	Weibo	110	22.0
	Douyin	80	16.0
Check‐in behavior	Frequent users	290	58.0
	Occasional users	160	32.0
	Prospective users	50	10.0
Device used	Smartphone	435	87.0
	Tablet	40	8.0
	Desktop/Laptop	25	5.0
Marital status	Single	220	44.0
	Married	260	52.0
	Divorced/Widowed	20	4.0

### 4.1 Measurement Model Statistics

**FIGURE 2:**
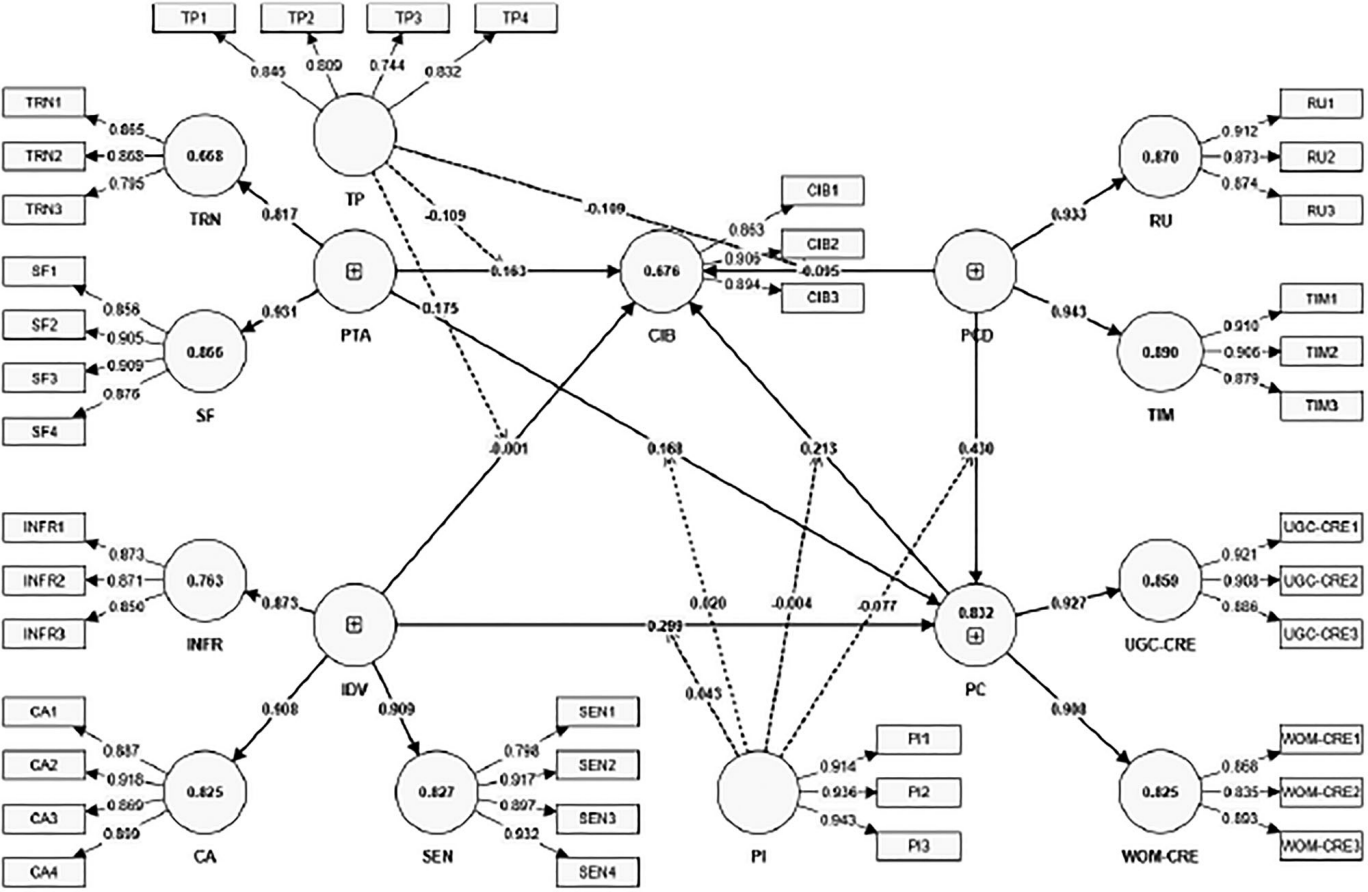
Measurement model.

The evaluation of the measurement model (Figure 2) demonstrates a high degree of psychometric robustness across all constructs, confirming the adequacy of the instrument for further structural testing. Outer loadings (Table 2) for all indicators exceed the recommended threshold of 0.70 (Hair et al. 2022), ranging from 0.744 to 0.945, which affirms that each item exhibits strong convergent validity and reliably reflects its underlying latent construct. Notably, high‐performing items such as TIM2 (0.906), PI2 (0.936), and CA2 (0.918) further reinforce the instrument's discriminative precision across cognitive, trust‐based, and social validation dimensions.

**TABLE 2:** Measurement model statistics.

Construct		Items	OL	VIF	CA	CR	AVE
First order	Second order						
CA		CA1	0.887	3.116	0.916	0.941	0.799
		CA2	0.918	3.706			
		CA3	0.869	2.475			
		CA4	0.899	2.935			
CIB		CIB1	0.863	1.954	0.865	0.917	0.788
		CIB2	0.906	2.612			
		CIB3	0.894	2.402			
	IDV	CA	0.896	2.427	0.880	0.926	0.807
		INFR	0.899	2.433			
		SEN	0.900	2.449			
INFR		INFR1	0.873	1.937	0.831	0.899	0.748
		INFR2	0.871	1.945			
		INFR3	0.850	1.856			
	PC	UGC‐CRE	0.923	1.883	0.813	0.914	0.842
		WOM‐CRE	0.913	1.883			
	PCD	RU	0.930	2.367	0.864	0.936	0.880
		TIM	0.945	2.367			
PI		PI1	0.914	2.956	0.923	0.951	0.867
		PI2	0.936	3.816			
		PI3	0.943	4.097			
	PTA	SF	0.893	1.433	0.710	0.873	0.775
		TRN	0.867	1.433			
RU		RU1	0.912	2.681	0.863	0.917	0.786
		RU2	0.873	2.069			
		RU3	0.874	2.179			
SEN		SEN1	0.798	1.864	0.909	0.937	0.788
		SEN2	0.917	3.794			
		SEN3	0.897	3.128			
		SEN4	0.932	4.256			
SF		SF1	0.856	2.322	0.909	0.936	0.786
		SF2	0.905	3.251			
		SF3	0.909	3.377			
		SF4	0.876	2.580			
TIM		TIM1	0.910	2.727	0.881	0.926	0.807
		TIM2	0.906	2.615			
		TIM3	0.879	2.172			
TP		TP1	0.845	2.011	0.822	0.883	0.654
		TP2	0.809	1.812			
		TP3	0.744	1.432			
		TP4	0.832	1.920			
TRN		TRN1	0.865	1.843	0.796	0.880	0.711
		TRN2	0.868	1.915			
		TRN3	0.795	1.499			
UGC‐CRE		UGC‐CRE1	0.921	2.955	0.889	0.931	0.819
		UGC‐CRE2	0.908	2.718			
		UGC‐CRE3	0.886	2.305			
WOM‐CRE		WOM‐CRE1	0.868	2.207	0.833	0.900	0.750
		WOM‐CRE2	0.835	1.626			
		WOM‐CRE3	0.893	2.446			

Abbreviations: CIB, check‐in behavior; IDV, information density value; INFR, information richness; PC, perceived credibility; PCD, peer consensus dynamic; PI, peer influence; PTA, personal trust anchor; TP, trust in platform.

Internal consistency reliability is well established. Composite reliability (CR) scores range between 0.883 and 0.951, comfortably above the 0.70 benchmark, whereas Cronbach's *α* values fall between 0.710 and 0.923. These metrics underscore excellent internal coherence and minimal measurement error, particularly for higher order constructs such as PI (CR = 0.951; *α* = 0.923) and credibility (CR = 0.914; *α* = 0.813). These findings provide strong statistical evidence that the constructs consistently capture stable dimensions across multiple items.

Convergent validity is confirmed through Average Variance Extracted (AVE) values, all of which exceed the 0.50 threshold, ranging from 0.654 (TP) to 0.867 (PI). The relatively lower AVE for TP reflects the construct's multidimensional nature—spanning security, reliability, and transparency—while still meeting the minimum acceptable level. Importantly, all second‐order constructs demonstrate high loading values from their respective subdimensions: IDV's loadings range from 0.896 to 0.900, and PCD is represented by Relevance to User (0.930) and Timeliness (0.945), both suggesting strong construct‐formative linkages.

Discriminant validity is strongly supported through the HTMT criterion (Table 3), with all inter‐construct correlations falling below the conservative 0.85 threshold. For instance, the HTMT between TP and PI is 0.551, and between CIB and WOM‐CRE is 0.836, confirming that conceptually adjacent constructs are empirically distinct. This is particularly critical in a model with theoretically overlapping domains such as credibility, trust, and social validation.

**TABLE 3:** Discriminant validity (HTMT).

Sl. no.	Construct	1	2	3	4	5	6	7	8	9	10	11	12
1.	CA												
2.	CIB	0.705											
3.	INFR	0.807	0.737										
4.	PI	0.488	0.513	0.475									
5.	RU	0.665	0.737	0.704	0.458								
6.	SEN	0.776	0.718	0.816	0.443	0.765							
7.	SF	0.763	0.712	0.830	0.486	0.715	0.731						
8.	TIM	0.820	0.819	0.829	0.474	0.811	0.848	0.808					
9.	TP	0.817	0.836	0.809	0.551	0.815	0.826	0.804	0.802				
10.	TRN	0.712	0.749	0.809	0.508	0.565	0.653	0.645	0.745	0.810			
11.	UGC‐CRE	0.808	0.780	0.810	0.428	0.813	0.828	0.815	0.847	0.816	0.705		
12.	WOM‐CRE	0.719	0.836	0.765	0.703	0.802	0.772	0.738	0.830	0.832	0.692	0.791	

Abbreviations: CIB, check‐in behavior; INFR, information richness; PI, peer influence; TP, trust in platform.

Multicollinearity was not an issue, as all variance inflation factor (VIF) values fall below the common threshold of 5, indicating that collinearity among indicators is minimal and will not bias the structural model estimates.

The inclusion of second‐order constructs enhances the theoretical depth and empirical parsimony of the model as suggested by Duarte and Amaro (2018) and Crocetta et al. (2021). For instant, IDV—operationalized through CA, INFR, and sentiment tone (SEN)—offers a multidimensional lens on cognitive elaboration. Likewise, PCD, comprising relevance to user and timeliness, captures dynamic social alignment cues.

### 4.2 Out‐of‐Sample Predictive Relevance (PLSpredict)

The PLSpredict procedure was conducted to assess the model's out‐of‐sample predictive performance, focusing on CIB. Table 4 presents item‐level predictive metrics for each CIB indicator, including *Q* ^2^ predict values and RMSE comparisons between the PLS‐SEM model and a linear regression (LM) benchmark (Danks et al. 1987; Legate et al. 2023).

**TABLE 4:** Model fit and predict.

Indicator	*Q* ^2^ predict	PLS‐SEM_RMSE	LM_RMSE	IA_RMSE
**CIB1**	0.486	0.761	0.743	1.062
**CIB2**	0.488	0.713	0.740	0.996
**CIB3**	0.537	0.649	0.666	0.954

Abbreviations: CIB, check‐in behavior; LM, linear regression; PLS‐SEM, partial least squares structural equation modeling.

All *Q* ^2^ predict values are above zero, indicating medium‐to‐strong predictive relevance (Legate et al. 2023). Although PLS‐SEM RMSE values are slightly higher than LM RMSE for CIB1 and CIB2, they are lower for CIB3, suggesting comparative or superior predictive accuracy for that item. The IA RMSE values reflect expected inference error under uncertainty but remain within an acceptable range.

### 4.3 Hypothesis Testing and Discussion

**FIGURE 3:**
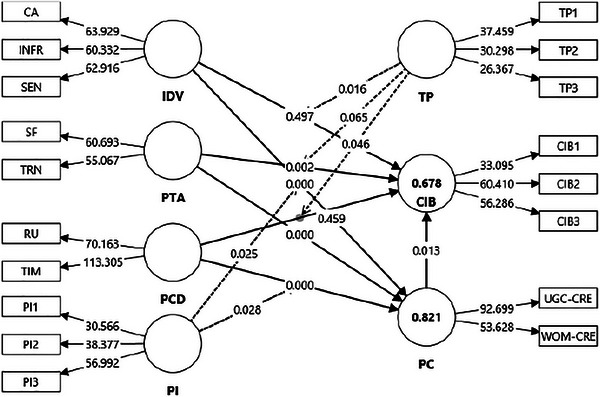
Structural model.

The structural model (Figure 3) provides detailed insights into the intricate dynamics underlying user CIB (CIB), achieving strong explanatory power with an *R* ^2^ of 0.678 (Table 5), which substantially exceeds the recommended threshold for robust behavioral research (Hair et al. 2022). Similarly, the exceptionally high explanatory capacity for PC (*R* ^2^ = 0.821) further underscores the theoretical importance of credibility as a mediating cognitive construct within digital engagement frameworks.

**TABLE 5:** Structural model statistics.

Hypothesis path		Std. beta	Std. div.	*t* values	*p* values	*f* ^2^	*r* ^2^	PCI LL	PCI UL	Support
H1a	PTA → CIB	0.203	0.069	2.934	<0.001	0.034	0.678	0.086	0.315	Yes
H1b	IDV → CIB	−0.001	0.122	0.008	0.497	0.000		−0.188	0.215	No
H1c	PC → CIB	0.248	0.111	2.229	0.013	0.031		0.056	0.422	Yes
H2a	PTA → PC	0.187	0.049	3.825	<0.001	0.054	0.821	0.109	0.270	Yes
H2b	IDV → PC	0.271	0.063	4.329	<0.001	0.070		0.166	0.370	Yes
H2c	PCD → PC	0.530	0.523	6.915	<0.001	0.258		0.409	0.661	Yes
H3a	TP × PTA → CIB	−0.084	0.056	1.513	0.065	0.008		−0.172	0.008	No
H3b	TP × IDV → CIB	0.156	0.073	2.147	0.016	0.020		0.040	0.279	Yes
H3c	TP × PCD → CIB	−0.104	0.062	1.681	0.046	0.011		−0.213	−0.010	No
H4a	PI × IDV → PC	−0.098	0.050	1.954	0.025	0.017		−0.187	−0.022	No
H4b	PI × PCD → PC	0.107	0.056	1.906	0.028	0.027		0.020	0.201	Yes
H5a	PTA → PC → CIB	0.046	0.024	1.957	0.025			0.014	0.092	Yes
H5b	IDV → PC → CIB	0.067	0.037	1.826	0.034			0.014	0.133	Yes
H5c	PCD → PC → CIB	0.132	0.061	2.165	0.015			0.039	0.239	Yes

*Note*: Significance threshold of <0.05 (one‐tailed) and 95% confidence intervals that do not include zero. Structural paths were estimated using 10,000‐sample bootstrapping in SmartPLS. Interaction terms reflect two‐stage moderation modeling. Mediation effects represent specific indirect effects tested via bootstrapping.

Abbreviations: CIB, check‐in behavior; IDV, information density value; PC, perceived credibility; PCD, peer consensus dynamic; PI, peer influence; PTA, personal trust anchor; TP, trust in platform.

PTAs significantly predict CIB (*β* = 0.203, *p* < 0.001, 95% CI [0.086, 0.315]). This finding corroborates existing trust theories (Mayer et al. 1995; Yan et al. 2024) that emphasize the critical role of personalized familiarity cues in reducing uncertainty and enhancing behavioral intentions. The observed modest effect size (*f* ^2^ = 0.034) indicates a meaningful yet limited direct influence, implying that PTA, whereas significant, functions best within a broader network of reinforcing factors. This interpretation aligns with findings by Akhtar et al. (2024), suggesting trust markers such as verified identities likely complement other mechanisms rather than independently driving substantial behavior changes.

In contrast, IDV failed to directly influence CIB (*β* = −0.001, *p* = 0.497, 95% CI [−0.188, 0.215]), diverging notably from expectations grounded in content quality theories (Batumalai 2023; Zhang et al. 2023). The negligible effect size (*f* ^2^ = 0.000) reinforces this non‐significant outcome. A critical interpretation suggests that dense, high‐quality information alone may not suffice to trigger user engagement, potentially due to users prioritizing trust‐based or social validation cues over purely informational ones. Consistent with the ELM (Petty and Cacioppo 1986), peripheral trust signals may overshadow central informational cues in decision‐making contexts involving social media check‐ins.

PC, however, significantly predicts CIB (*β* = 0.248, *p* = 0.013, 95% CI [0.056, 0.422]), albeit with a moderate effect size (*f* ^2^ = 0.031). This finding validates credibility's role as a crucial cognitive determinant of user behavior (Sarkar et al. 2019; Zhu, Lin, et al. 2020). Nonetheless, the moderate effect indicates credibility may be necessary but insufficient on its own to guarantee behavior change, such as emotional resonance or perceived value, to more effectively leverage credibility's behavioral potential.

The antecedents to PC demonstrate considerable explanatory strength (*R* ^2^ = 0.821), especially through PCDs, which exerted an unexpectedly strong positive effect (*β* = 0.530, *p* < 0.001, 95% CI [0.409, 0.661]) with a substantial effect size (*f* ^2^ = 0.258). This robust finding challenges previous assumptions that peer consensus may have only peripheral effects on credibility (Eg et al. 2023), emphasizing instead that social validation critically shapes credibility judgments. Meanwhile, IDV (*β* = 0.271, *p* < 0.001, 95% CI [0.166, 0.370]; *f* ^2^ = 0.070) and PTA (*β* = 0.187, *p* < 0.001, 95% CI [0.109, 0.270]; *f* ^2^ = 0.054) both significantly influenced PC, though with comparatively moderate strengths. This underscores the interplay among multiple cues—social validation, content richness, and trustworthiness—in shaping credibility, highlighting an opportunity for future research to investigate boundary conditions and hierarchical influences among these credibility determinants.

**FIGURE A1:**
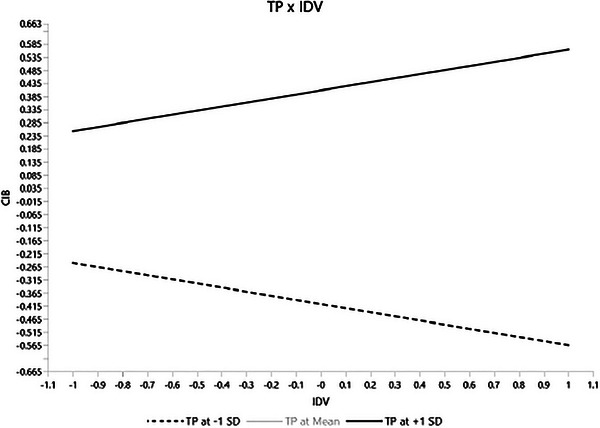
Interaction effect of trust in platform (TP) and information density value (IDV) on check‐in behavior (CIB) (corresponds to Hypothesis H3b).

Moderation analyses further enrich the nuanced understanding of the dynamics underlying CIB, revealing complexities that traditional theories have not fully captured. TP significantly and positively moderates the relationship between IDV and CIB (*β* = 0.156, *p* = 0.016, 95% CI [0.040, 0.279], *f* ^2^ = 0.020, Figure A1). This finding corroborates institutional trust theories positing that platform‐level assurances, such as robust security features, transparent ratings, or verified user guidelines, amplify users’ responsiveness to high‐quality informational content (Abramova et al. 2017). Critically, however, the relatively modest effect size suggests that although institutional trust can enhance content‐driven behavior, this mechanism alone may be insufficient without additional cognitive or emotional support.

**FIGURE A2:**
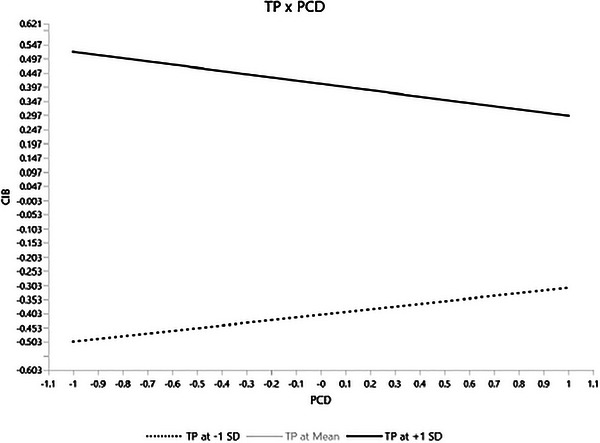
Interaction effect of trust in platform (TP) and peer consensus dynamics (PCDs) on check‐in behavior (CIB) (corresponds to Hypothesis H3c).

Conversely, TP negatively moderates the effect of PCDs on CIB (*β* = −0.104, *p* = 0.046, 95% CI [−0.213, −0.010], *f* ^2^ = 0.011, Figure A2). This inverse moderation offers intriguing implications, critically highlighting a potential trust‐substitution effect. High institutional trust appears to diminish users’ reliance on peer‐based consensus cues, implying that once institutional assurances are adequately perceived, users may no longer depend heavily on social proof for behavioral validation (Otterbring and Folwarczny 2024; Pérez López et al. 2025). However, the small effect size demands cautious interpretation: It suggests only a subtle reduction in social dependency, leaving open the possibility that peer consensus remains influential for subsets of users who inherently value social validation over institutional guarantees.

**FIGURE A3:**
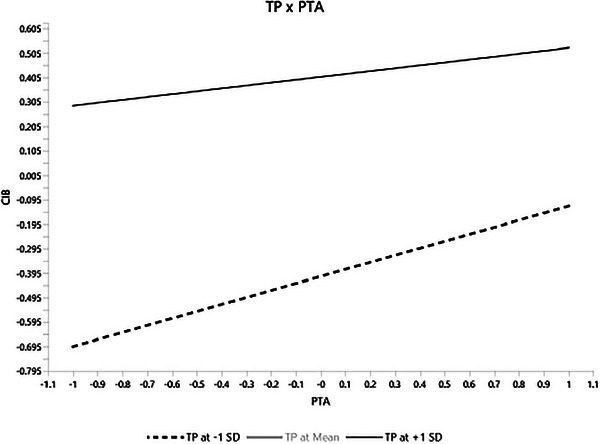
Interaction effect of trust in platform (TP) and personal trust anchors (PTAs) on check‐in behavior (CIB) (corresponds to Hypothesis H3a).

The non‐significant moderation of TP on the PTAs → CIB path (*β* = −0.084, *p* = 0.065, 95% CI [−0.172, 0.008], *f* ^2^ = 0.008, Figure A3) provides important insights despite its lack of statistical significance. The negligible moderation effect critically suggests that interpersonal trust formed through personalized trust anchors operates independently of institutional trust mechanisms. This result, though unexpected from trust‐transfer perspectives (Ahmed and Aziz 2024; Mao et al. 2020), implies complexity in trust formation, where personal connections might establish a separate cognitive pathway distinct from institutional assurances. This nuanced finding warrants further critical exploration into conditions under which platform‐level trust and PTAs intersect or diverge, possibly contingent on trust violation histories or platform reputation variations.

**FIGURE A4:**
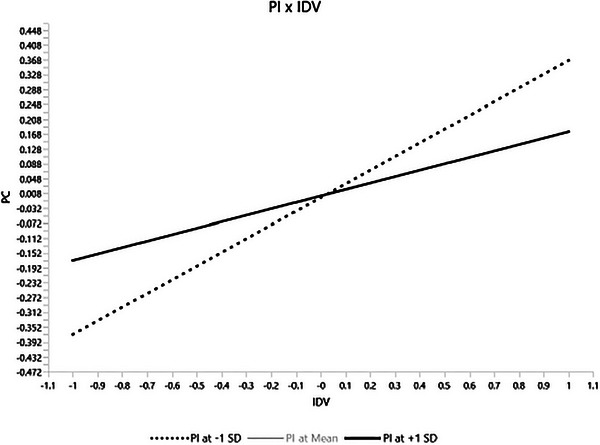
Interaction effect of peer influence (PI) and information density value (IDV) on perceived credibility (PC) (corresponds to Hypothesis H4a).

Similarly, PI displays intriguing complexity through its moderation effects. Contrary to initial hypotheses, PI negatively moderates the relationship between IDV and PC (*β* = −0.098, *p* = 0.025, 95% CI [−0.187, −0.022], *f* ^2^ = 0.017, Figure A4). Critically interpreted, this suggests that heightened peer scrutiny or intensified social interaction may inadvertently provoke skepticism toward content that appears overly detailed or sophisticated. This finding critically challenges conventional assumptions about PI uniformly reinforcing informational credibility, aligning instead with reactance theories and cognitive overload frameworks (Turel and Osatuyi 2021). It raises important questions regarding the effectiveness of rich content in contexts of high peer pressure or extensive peer commentary, indicating that platforms should carefully manage social interactions to avoid diluting perceived informational authenticity.

**FIGURE A5:**
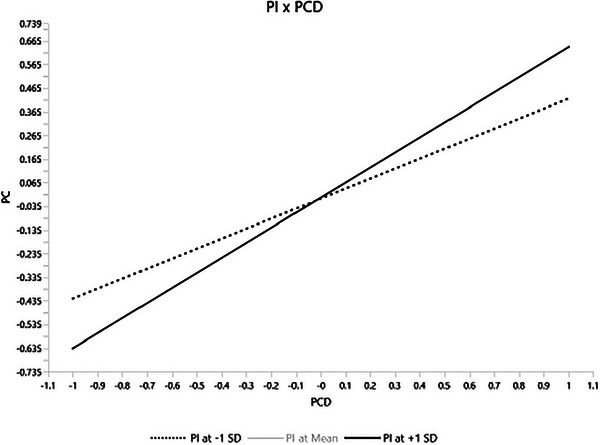
Interaction effect of peer influence (PI) and peer consensus dynamics (PCDs) on perceived credibility (PC) (corresponds to Hypothesis H4b).

In contrast, PI positively moderates the PCD–PC relationship (*β* = 0.107, *p* = 0.028, 95% CI [0.020, 0.201], *f* ^2^ = 0.027, Figure A5), supporting bandwagon and social validation theories (Koranteng et al. 2022). This outcome reinforces the critical understanding that peer consensus cues gain additional credibility strength under strong social influence, particularly within highly cohesive or collectivist contexts. Critically, however, the moderate effect size suggests limitations in generalizability, implying that peer consensus significantly shapes credibility judgments predominantly under conditions of social ambiguity or risk.

Lastly, the mediation analyses significantly enrich understanding of the credibility construct's intermediary role. The findings confirm that PC significantly mediates all three pathways: PTA → PC → CIB (*β* = 0.046, *p* = 0.025, 95% CI [0.014, 0.092]), IDV → PC → CIB (*β* = 0.067, *p* = 0.034, 95% CI [0.014, 0.133]), and, notably stronger, PCD → PC → CIB (*β* = 0.132, *p* = 0.015, 95% CI [0.039, 0.239]). These mediation results critically affirm PC's function in transforming cognitive appraisals into behavioral intentions, consistent with expectancy‐value theory (Reiss 1991; Zhu, Lin, et al. 2020). Notably, the more substantial mediation via PCD reveals social consensus as a potent credibility‐building mechanism that strongly motivates behavior. Critically, however, the modest mediation effects observed for PTA and IDV suggest partial rather than comprehensive mediation, indicating PC may share explanatory power with alternative psychological constructs.

In sum, the integrative analysis reveals a sophisticated picture where trust, informational richness, peer consensus, and credibility intertwine with varying strengths, conditionalities, and interactions. The high explanatory power indicated by *R* ^2^, alongside careful inspection of effect sizes and confidence intervals, strengthens confidence in these interpretations while simultaneously highlighting important avenues for further empirical inquiry.

## 5 Implications of This Study

### 5.1 Theoretical Implications

This study makes significant theoretical contributions by advancing and integrating Trust Theory, Social Influence Theory, and the ELM in the context of digital user engagement. It offers a multi‐layered theoretical lens to understand how interpersonal trust, institutional trust, peer dynamics, and information quality interact to shape credibility perceptions and behavioral outcomes in online environments. Rather than reinforcing existing frameworks, the findings challenge core assumptions, propose boundary conditions, and introduce new theoretical pathways relevant to digital behavior research.

Within Trust Theory, the study foregrounds the coexistence and partial independence of interpersonal and institutional trust in influencing online behavior. Traditionally conceptualized as mutually reinforcing, this research suggests these two trust types may function in parallel but distinct roles. Interpersonal trust—rooted in familiarity, credibility cues, and identity signals—operates as a cognitive filter for evaluating content and initiating behavior. Institutional trust, on the other hand, plays a contextual amplifying or substitutive role, enhancing the effect of content or compensating for weak peer consensus. This layered structure reveals that trust in digital environments is non‐linear and contingent, refining simplistic models of trust transfer. It invites theorists to reconsider assumptions that trust sources are additive and instead conceptualize trust as a strategically allocated cognitive resource in environments of information overload and social complexity.

In relation to Social Influence Theory, the study offers a more nuanced interpretation of how peer dynamics shape credibility and decision‐making. Rather than assuming social validation is universally beneficial, the findings reveal a dual‐process mechanism: Although moderate peer consensus enhances credibility judgments, excessive social uniformity may trigger skepticism, cognitive resistance, or heuristic fatigue. This challenges the notion of PI as uniformly persuasive and calls for a threshold‐based view of social validation, where the effectiveness of peer cues is dependent on volume, diversity, and user sensitivity to conformity. Additionally, the study situates PI as both a moderator and a mediator of cognitive appraisals, extending social influence theory to include interactional and conditional functions rather than unidirectional persuasion. This repositioning aligns social validation more closely with complex information‐processing environments and strengthens its explanatory power for behaviors shaped by algorithmically curated or socially saturated media.

The ELM is significantly extended by the study's demonstration of how central and peripheral routes to persuasion function in concert rather than isolation. The findings suggest that INFR alone is insufficient to produce behavioral outcomes unless supported by peripheral cues like trust or peer validation. This undermines the classical ELM bifurcation and encourages a dynamic dual‐processing model, wherein central and peripheral cues interact continuously, often non‐linearly, in shaping perceptions and behaviors. Moreover, the influence of contextual factors—such as the credibility of the source or the structure of peer input—suggests that processing route selection is not purely a function of user motivation and ability but also of environmental complexity and cognitive overload. This invites a critical rethinking of ELM in digital contexts, where information is abundant but attention is scarce, and where social dynamics and algorithmic cues constantly shift users’ processing orientation.

Integrating these theoretical advancements, the study proposes a multi‐cue, multi‐level model of digital engagement. It conceptualizes trust as a foundational gatekeeper, social influence as a contextual modulator, and INFR as a cognitive catalyst—each functioning within a system of mutual dependency and occasional compensation. This layered framework advances theory by shifting focus from static cause‐effect linkages to adaptive configurations of cognitive, social, and institutional inputs. Importantly, it moves beyond the siloed application of single theories to suggest that behavioral outcomes in digital environments are best understood through the interaction of trust‐building, information processing, and social signaling mechanisms.

In sum, this study's theoretical contributions lie in its ability to critique, synthesize, and extend foundational theories in light of emerging digital behavior. It calls for scholars to abandon linear, additive, and context‐independent models in favor of relational, conditional, and dynamic theoretical architectures—ones that reflect the complexity, saturation, and socially embedded nature of contemporary online interactions.

### 5.2 Practical Implications

This study provides critical guidance for platform designers, digital marketers, and policymakers in China seeking to enhance user engagement through trust‐building, social validation, and content credibility in its rapidly evolving digital ecosystems. It underscores that behavioral engagement is not solely a function of information quality but of how trust and peer dynamics are embedded into users’ interaction journeys—especially relevant in the collectivist and surveillance–conscious context of Chinese digital culture.

The influence of PTAs on both behavior and credibility reinforces the need for Chinese platforms to implement mechanisms that foster identity continuity and transparency in ways that align with user expectations around relational trust. Features like verified real‐name profiles, visible transaction histories, and trust indicators (e.g., Zhima Credit on Alipay) serve as crucial heuristics in navigating uncertainty. In a market where users are wary of counterfeit information or state overreach, consistency and familiarity become indispensable in driving engagement.

The nuanced role of IDV reveals that rich, authentic content—such as detailed reviews or long‐form video explanations common on Xiaohongshu—may not suffice to trigger action unless supported by relational cues or social proof. Platforms should thus curate content that is emotionally resonant and socially situated, embedding narrative depth and multimedia personalization. Content must not only be informative but also culturally relatable and moderated in‐line with both user expectations and state guidelines to preserve perceived safety and authenticity.

Findings around PI challenge assumptions about the uniformly positive role of social validation, particularly in a culture where conformity pressures are high. Although moderate peer endorsement through likes or danmu (live comment overlays) can elevate credibility, excessive consensus—especially when algorithmically inflated—can trigger user skepticism or disengagement. Platforms should design social layers that surface diverse opinions, counteract information silos, and protect against viral manipulation. This is particularly important for sensitive domains like health or education, where critical thinking is vital.

The amplification role of TP emphasizes that institutional assurances—particularly around privacy, censorship transparency, and platform reliability—are critical for user action in China's tightly regulated environment. Features such as privacy dashboards, AI transparency notices, and timely system updates can foster perceived procedural fairness. Importantly, platform trust can reduce users’ reliance on peer consensus, allowing them to act more independently—particularly relevant in risk‐averse user segments such as older adults or first‐time rural internet users.

The limited yet significant mediating role of PC highlights that while credibility is vital, it alone does not bridge the perception–action gap. Platforms must supplement credibility with emotional and motivational cues, including context‐aware nudges (e.g., “others near you are checking in”), interactive features, and recommendation systems tailored via local cultural insights. Given that Chinese consumers often weigh both affective resonance and utility, combining credible content with emotionally intelligent design is crucial to stimulate behavioral engagement.

For marketers and content strategists, the study signals a shift from optimization for exposure to optimization for relational trust and cultural relevance. Content strategies should emphasize value congruence, dialectal tone, and identity signaling. Collaborating with micro‐KOLs (key opinion leaders) who reflect users’ everyday realities—rather than top‐tier celebrities—can yield more authentic and persuasive engagement in vertical platforms like Douyin or Bilibili.

From a policy perspective, the findings support the call for trust‐centric regulation in China's platform economy. While existing laws such as the PIPL and CAC content directives mandate certain safeguards, additional frameworks could enhance algorithmic transparency, clarify trust signal standards, and encourage ethical nudging. Regulatory innovation must balance user protection, platform autonomy, and public interest, reinforcing digital ecosystems that are not only secure but also participatory and psychologically safe.

Overall, the study calls for a systems‐level recalibration of digital engagement strategies in China. Trust, PI, and INFR must be dynamically integrated through culturally attuned design, user empowerment, and institutional accountability. Platforms that embed trust into both technology and social interaction will be best equipped to foster resilient engagement across China's diverse and rapidly maturing digital public sphere.

### 5.3 Conclusion and Future Research Directions

This study provides a cohesive analysis of user behavior in digital environments by foregrounding the interrelated roles of trust, PI, and cognitive processing. The significant effect of PTAs on both CIB and PC reaffirms the importance of familiarity, transparency, and identity consistency as foundational dimensions of trust—especially within Chinese platforms where rapid information exchange coexists with uncertainty and institutional scrutiny. Even in transient or algorithmically curated interactions, the persistence of interpersonal trust cues underscores their enduring behavioral relevance. Meanwhile, constructs like IDV and PCDs influence PC but show contingent effects on behavior, dependent on whether they are reinforced or moderated by trust mechanisms or social validation. These findings expand our understanding of the layered pathways through which users make decisions in culturally nuanced, data‐rich environments.

The theoretical contributions span Trust Theory, Social Influence Theory, and the ELM, emphasizing that trust and peer dynamics are neither static nor uniformly positive. The findings complicate conventional social influence frameworks by showing that over‐conformity in peer consensus may reduce credibility, while moderate social validation enhances it—particularly salient in collectivist digital cultures like China's, where social harmony and individual discernment coexist. ELM is refined by demonstrating how central‐route cues (e.g., content richness) require the support of peripheral cues (e.g., TP or peer endorsement) to influence behavior, revealing the interdependence of rational and heuristic processing in high‐stakes digital interactions.

Future research should further explore how trust and PI unfold across different digital contexts and cultural settings. In China's fast‐changing digital landscape—shaped by platform governance, state regulation, and evolving user expectations—there is a growing need to examine how users develop and sustain trust over time. Longitudinal designs could reveal how credibility judgments evolve as users gain experience, while cross‐cultural comparisons could clarify which trust cues are universally effective versus locally contingent. Emotional and affective factors—such as awe, anxiety, or pride—could also be incorporated to expand the cognitive‐social framework used here. Moreover, the impact of emerging technologies such as emotionally intelligent AI, algorithmic transparency tools, or AR‐based interactions on trust and engagement offers fertile ground for innovation‐driven research.

Further inquiry is also needed into the tipping points at which peer validation becomes counterproductive or when institutional trust can substitute for social proof. Experimental or multi‐wave designs could illuminate the causal mechanisms behind these tipping points and shed light on how users navigate conflicting cues. Applying the model to varied behavioral domains—such as digital health compliance, e‐governance participation, or educational technology adoption—could extend the generalizability and application of these findings across sectors.

Policy and ethical considerations remain central to these discussions. In an era of increasing algorithmic mediation and social engineering, regulators and platform architects must ensure that trust‐building mechanisms do not inadvertently amplify misinformation or erode user autonomy. Chinese digital platforms, in particular, must navigate the dual imperatives of regulatory compliance and user‐centered trust. Designing for transparency, respecting user agency, and embedding ethical nudges into system architectures will be key to fostering digital ecosystems that are both innovative and trustworthy.

In conclusion, this study advances theoretical and practical understanding of digital behavior by demonstrating how trust, peer dynamics, and information processing intersect to shape engagement. These insights serve as a foundation for future research and offer actionable guidance for practitioners and policymakers seeking to create more credible, resilient, and user‐aligned digital environments—particularly in contexts like China where trust is both a cultural imperative and a strategic asset.

## Author Contributions

**Xiaoshuang Lu**: writing – original draft, writing – review and editing, investigation. **Kavitha Balakrishnan**: methodology, conceptualization, supervision. **Tak Jie Chan**: formal analysis, validation. **Meng Na**: project administration, resources, software.

## Ethics Statement

Ethical approval was obtained from Multimedia University, Cyberjaya, on fourth November 2024, and reference no. RMC/REC/EA/088/2024.

## Consent

Oral consent was obtained from all individuals involved in this study.

## Conflicts of Interest

The authors declare no conflicts of interest.

## Peer Review

The peer review history for this article is available at https://publons.com/publon/10.1002/brb3.70887.

## Data Availability

The data that support the findings of this study are available from the corresponding author upon reasonable request.

## Appendix A Measurement Items

### A.1

**Table:**

Construct	Indicator	Item	Source
Source familiarity (SF)	SF1	The source of this information feels familiar to me	Komiak and Benbasat (2006)
	SF2	I recognize this source as trustworthy	
	SF3	The source of this information is someone I frequently encounter online	
	SF4	I feel comfortable relying on this source	
Transparency (TRN)	TRN1	The source of this information is open about its intentions	Mao et al. (2020)
	TRN2	I can easily verify the claims made by this source	
	TRN3	This source provides complete and honest information	
Consistency (CA)	CA1	The source has been consistent in the quality of its information	Mcknight et al. (2011)
	CA2	I have never encountered contradictory information from this source	
	CA3	The source's information aligns with my expectations	
	CA4	I find this source reliable over time	
Information richness (INFR)	INFR1	The content provides detailed and comprehensive information	Zhang et al. (2023)
	INFR2	This information is well‐structured and easy to follow	
	INFR3	The content includes various types of media (e.g., text, images, videos)	
Content authenticity (UGC‐CRE)	UGC‐CRE1	This content seems genuine and not fabricated	Zhu, Li et al. (2020)
	UGC‐CRE2	The creator's intentions appear sincere	
	UGC‐CRE3	The content reflects real experiences	
Sentiment tone (SEN)	SEN1	The tone of this information conveys a balanced perspective	Wang et al. (2023)
	SEN2	This information uses language that evokes trust	
	SEN3	The sentiment in the content aligns with my values	
	SEN4	The emotional tone of this information influences my perceptions positively	
Relevance to user (RU)	RU1	This information is highly relevant to my interests	Sarkar et al. (2019)
	RU2	The content resonates with my personal needs	
	RU3	I find the information aligns with my preferences	
Timeliness (TIM)	TIM1	The information is current and up to date	Sarkar et al. (2019)
	TIM2	The timing of this content makes it more useful to me	
	TIM3	The content is relevant to recent developments or trends	
eWOM credibility (WOM‐CRE)	WOM‐CRE1	The electronic word‐of‐mouth content appears reliable	Wang et al. (2023)
	WOM‐CRE2	I find the reviews shared online credible	
	WOM‐CRE3	Online recommendations influence my decisions positively	
Check‐in behavior (CIB)	CIB1	I frequently engage in check‐in behavior on social media platforms	Luarn et al. (2015)
	CIB2	I am likely to share my location via check‐ins in the future	
	CIB3	Check‐in features influence my platform usage	
Trust in platform (TP)	TP1	I trust this platform to handle my data securely	Mao et al. (2020)
	TP2	This platform ensures the accuracy of the information it provides	
	TP3	The platform is transparent about its policies	
	TP4	The platform is reliable in providing consistent user experiences	
Peer influence (PI)	PI1	My peers' actions influence my behavior on this platform	Reinikainen et al. (2020)
	PI2	I value my peers' opinions about using check‐in features	
	PI3	Peer feedback affects how I engage with platform content	
